# Emergence of OXA-48-producing *Klebsiella pneumoniae* ST5994, a sister lineage of the high-risk ST307 clone

**DOI:** 10.3389/fcimb.2026.1872766

**Published:** 2026-09-29

**Authors:** Jorge Rodríguez-Grande, Domingo Fernández-Vecilla, Rosalía Palomino-Cabrera, Samuel García-García, Daniela Vallejo-Iriarte, Nuria Valcárcel-Fraile, María Siller-Ruíz, María Carmen Fariñas, María Pía Roiz Mesones, Jorge Calvo-Montes, Alain Ocampo-Sosa, Zaira Moure, Sergio García-Fernández

**Affiliations:** 1 Servicio de Microbiología, Hospital Universitario Marqués de Valdecilla-Instituto de Investigación Marqués de Valdecilla (IDIVAL), Santander, Spain; 2 Consorcio de Investigación Biomédica en Red (CIBER) de Enfermedades Infecciosas, Instituto de Salud Carlos III, Madrid, Spain; 3 Servicio de Enfermedades Infecciosas, Hospital Universitario Marqués de Valdecilla-Instituto de Investigación Marqués de Valdecilla (IDIVAL), Santander, Spain; 4 Departamento de Biología Molecular, Universidad de Cantabria, Santander, Spain

**Keywords:** antimicrobial resistance, carbapenemase-producing *Klebsiella pneumoniae*, FT-IR spectroscopy, molecular epidemiology, OXA-48, ST307, ST5994, whole-genome sequencing

## Abstract

**Introduction:**

Carbapenemase-producing *Klebsiella pneumoniae* (CP-KP) represents a major public health threat. The global dissemination of antimicrobial resistance is largely driven by high-risk clones (HRCs), such as ST307/OXA-48, ST11/OXA-48 and ST512/KPC-3, which currently dominate the epidemiology of CP-KP in Spain. This study aimed to describe the emergence, dissemination and genomic evolution of an OXA-48-producing *K. pneumoniae* ST5994 clone in Cantabria, Spain, and to characterize its genomic, phenotypic and epidemiological features.

**Methods:**

A retrospective genomic surveillance study of CP-KP isolates was conducted in Cantabria from 2021 to 2024. Whole-genome sequencing was used to characterize sequence types, resistome, mobilome, virulome, pangenome and phylogenetic relationships. Antimicrobial susceptibility and biofilm production assays were performed. Fourier-transform infrared (FT-IR) spectroscopy combined with machine learning algorithms was used to develop a rapid method for HRCs discrimination.

**Results:**

Among 448 non-duplicate CP-KP isolates, *bla*_OXA-48_ was the predominant carbapenemase. *K. pneumoniae* ST5994 was first detected in the second quarter of 2023 and rapidly became one of the dominant CP-KP clones at both reference hospital and regional levels over a two-year period, displacing previously prevalent HRCs, such as *K. pneumoniae* ST11 and *K. pneumoniae* ST307. The newly identified clone harbored two potentially conjugative plasmids carrying *bla*_OXA-48_ carbapenemase and *bla*_CTX-M-15_ extended-spectrum beta-lactamase. At the time of writing, eight ST5994 genomes were publicly available, all from the Americas and none carrying a *bla*_OXA-48_ gene, therefore this study represents both the first description of this ST in Europe and its first association with OXA-48. Genomic and epidemiological analyses were compatible with sustained dissemination within the regional healthcare network, although the specific transmission pathways could not be established with the available data. Comparative analyses supported a close evolutionary relationship between *K. pneumoniae* ST307 and ST5994.

**Conclusions:**

Our findings highlight the rapid emergence and regional replacement capacity of the novel OXA-48-producing *K. pneumoniae* ST5994. The integration of genomic and phenotypic data provides insights into its evolutionary trajectory and transmission dynamics. Furthermore, the proposed FT-IR-based approach combined with machine learning offers a promising tool for rapid discrimination of HRCs, supporting surveillance and infection control strategies.

## 1 Introduction

Carbapenemase-producing Enterobacterales (CPE) have become a major threat for global healthcare (Tacconelli et al., 2018). Some clones have shown fast spread and prevalence, while carrying whole collections of antimicrobial resistance genes, giving rise to high-risk clones (HRCs). Although diverse, main carbapenemase-producing *Klebsiella pneumoniae* (CP-KP) clones worldwide (Arcari and Carattoli, 2023) carry either *bla*_KPC_ or *bla*_OXA-48_ or *bla*_NDM_ and belong to sequence types (ST) 11, 15, 147, 258 and 512, while in Europe (Campos-Madueno et al., 2022) and particularly in Spain (Budia-Silva et al., 2024), the CP-KP is dominated by the HRC ST307/OXA-48, ST11/OXA-48 and ST512/KPC-3 clones (Cañada-García et al., 2022).

In recent years, new *Klebsiella pneumoniae* multidrug-resistant lineages have emerged internationally. Among them, *K. pneumoniae* ST307 has been reported all around the globe (Castanheira et al., 2013) and acquired special prominence since its first description, during the early to mid-1990s (Peirano et al., 2020). At first, it was associated with extended-spectrum beta-lactamase *bla*_CTX-M-15_, but in the last years it has been found carrying *bla*_KPC-3_ and *bla*_OXA-48_ carbapenemase genes, raising concerns on their fast spread and antimicrobial resistance acquisition (Peirano et al., 2020).

Modern sequencing technologies allow clinical microbiologists to track in real time the spread of well-known pathogens and the emergence of risk clones, while assessing the genetic features of these pathogens, their mobile genetic elements (MGEs) and the antimicrobial resistance and virulence genes they carry.

The aim of this study was i) to perform a descriptive, retrospective study investigating the epidemiology of CP-KP in the Cantabria region of northern Spain, during a 3-year period (2021-2024), ii) to describe the emergence of the new OXA-48-producing *K. pneumoniae* ST5994 clone, iii) to compare *K. pneumoniae* ST5994 with the HRC *K. pneumoniae* ST307, in terms of antimicrobial susceptibility, mobilome, resistome, virulome and biofilm production and to establish whether ST5994 arose from within the local ST307 population or represents a separate lineage finally, iv) to elaborate a method for rapid detection and discrimination of these two CP-KP high-risk clones based on discrimination of Fourier-Transform Infrared (FT-IR) spectra using machine learning algorithms.

## 2 Materials and methods

### 2.1 Study design

A retrospective study was conducted from January-2021 to December-2024 to describe first the epidemiology of CP-KP in the Cantabria region, Spain. Following the Spanish Network of Microbiology Laboratories for the Surveillance of Antibiotic-Resistant Microorganisms (RedLabRA), every new case of infection or colonization of CPE is molecularly characterized (Cañada-García et al., 2021). This surveillance system has been in operation in Cantabria since January 2021. The health system in this region is made up of two secondary hospitals and one tertiary hospital, the latter being the reference for molecular characterization. Data in this study comprises patients from all these hospitals.

Information on dates and length of admission, hospitalization wards, and previous admissions was recovered from patients’ medical records. The ethical committee of Marqués de Valdecilla University Hospital approved the study (2024.145) and decided that informed consent was not necessary as the information would only be used to study hospital transmission. Finally, to characterize the emergence of the ST5994 lineage during its initial expansion, we selected the first 32 consecutive CP-KP ST5994 cases detected between June 2023 to June 2024, together with 33 of their related *K. pneumoniae* ST307 counterparts from the same period, in order to compare the genomes of the two lineages in terms of gene acquisition/loss, especially at mobilome, virulome and resistome level. One ST5994 isolate (#0498) was not successfully sequenced due to contamination, and was therefore excluded from all assembly-based analyses while being retained in the epidemiological and phenotypic description. Comparative genomic analyses were consequently performed on 31 K*. pneumoniae* ST5994 and 33 K*. pneumoniae* ST307 genomes.

### 2.2 Identification, antimicrobial susceptibility testing and carbapenemase detection

All bacterial isolates were identified by matrix-assisted laser desorption ionization-time of flight mass spectrometry (MALDI-TOF MS; Vitek MS, BioMérieux, France). Antimicrobial susceptibility testing (AST) was performed using Vitek-2 cards (BioMérieux, France) and Sensititre EUMDROXF panels (Thermo Fisher Scientific, United States). EUCAST v.16.0 breakpoints were applied (http://www.eucast.org/ (EUCAST et al., 2017)). Isolates suspected of carrying a carbapenemase (MICs higher than 0.125 mg/L for meropenem or ertapenem) were molecularly characterized using a multiplex PCR for detection of acquired carbapenemase genes (Poirel et al., 2011).

### 2.3 Biofilm production assay

Biofilm formation was evaluated by the crystal violet (CV) staining assay, following a previously described protocol (Díaz-Ríos et al., 2021) with slight modifications. Briefly, 100 μL of a 0.2 McFarland bacterial suspension were added to 900 μL of Mueller-Hinton broth (MHB; BD Difco, Thermo Fisher Scientific Inc., Waltham, MA, USA) in each well of a 24-well plate (Nunc, Thermo Fisher Scientific Inc., Waltham, MA, USA). Each strain was tested in triplicate. Plates were incubated overnight at 37 °C. After incubation, the supernatant containing planktonic bacteria was carefully removed, and adherent cells were stained with 0.7% CV solution (Sigma-Aldrich, St. Louis, MO, USA) for 12 minutes.

Unbound dye was eliminated by washing three times with sterile distilled water, and the retained CV was solubilized with 33% (v/v) acetic acid (Sigma-Aldrich, St. Louis, MO, USA). Absorbance was recorded at 600 nm using an Infinite^®^ microplate reader (Tecan Trading AG, Männedorf, Switzerland), with nine measurements per well after orbital shaking.

The entire experiment was carried out on three independent occasions. Biofilm production was classified according to four thresholds: non-producer (OD ≤ 0.05), weak (OD > 0.05–0.1), moderate (OD > 0.1–0.3), and strong biofilm producer (OD > 0.3). The OD values obtained for each strain were corrected by subtracting the blank (medium only) control (Díaz-Ríos et al., 2021).

### 2.4 Whole genome sequencing

Genomic DNA was extracted from bacterial isolates using DNEasy Blood&Tissue Kit (Qiagen, Hilden, Germany). Libraries were prepared using Oxford Nanopore Rapid Barcoding Kit (SQK-RBK 114-96) and sequenced in ONT MinION r9.4.1 FlowCells (Nanopore Technologies, Oxford, UK). All CPE were sequenced in batches of 10–16 samples every week for one year and further identified and analyzed.

### 2.5 Bioinformatics

#### 2.5.1 Preprocessing

Long reads were basecalled using Dorado basecaller v.7.3.11 ((c) 2024 Oxford Nanopore Technologies, PLC.) in super-accuracy mode, barcodes demultiplexed and reads filtered (length>300bp, Q>12) and quality controlled using NanoPack (De Coster and Rademakers, 2023) v.1.4.1. Kraken (Wood et al., 2019) v.2.1.3 with Kraken Standard database (January 2024 update) was used to check for contamination in long reads, thus, samples containing >10% reads assigned to taxa different to *Klebsiella pneumoniae* were discarded.

#### 2.5.2 Assembly

was performed using Trycycler (Wick et al., 2021) v.0.5.3 with two rounds of long-read error correction with medaka v.1.7.2 (^©^ 2018-Oxford Nanopore Technologies Ltd.). All chromosome assemblies were re-circularized using Circlator (Hunt et al., 2015) v.1.5.5 and the start of *dnaA* gene set to position zero. Genome assembly quality was checked out using Quast (Gurevich et al., 2013) v.5.2.0.

#### 2.5.3 Annotation

Genomes were typed using Gambit (Lumpe et al., 2023) v.1.0.0 for subspecies typing and MLST v.2.23 (https://github.com/tseemann/mlst), using the PubMLST database (Jolley et al., 2018) for sequence typing. *K. pneumoniae* sequences proven to belong to STs 307 and 5994 were chosen for further analysis. After that, their proteins were annotated using Bakta (Schwengers et al., 2021) v.1.9.2 with Bakta DataBase v.5.1. Antimicrobial resistance genes (AMR) were identified with ResFinder v4.7.2 (Bortolaia et al., 2020), database version 2.6.0 (thresholds: 90% identity, 75% coverage). Virulence genes including *rmpADC, rmpA2, ybt, clb, iuc* and *peg-344*, kleborate score and capsule types were analyzed using Kleborate (Lam et al., 2021) v.3.1.2 and checked out using BLASTn (Camacho et al., 2009)v.2.12.0+ while biofilm-associated genes including *mrkABCD* and *fimAH* were also assessed using BLASTn. The *fosA* alleles were additionally typed against the NCBI Reference Gene Catalog using AMRFinderPlus v.4.2.7 (database version 2026-05-15.1), RGI v.6.0.8 with CARD (Alcock et al., 2023) database v.4.0.1 and compared with catalogued *fosA* alleles by BLASTn. Other chromosomal determinants of fosfomycin susceptibility (*fosA*, *glpT*, *uhpT*, *uhpA*, *cyaA*, *ptsI* and *murA*) were compared between ST307 and ST5994 lineages.

#### 2.5.4 Mobile genetic elements

Plasmids and other MGEs were detected using MOB-suite (Robertson and Nash, 2018) v.3.0.3, Copla v.1.0 and Integron Finder (Néron et al., 2022) v.2.0.2. Plasmid maps were plotted using Proksee (Grant et al., 2023) v1.0.0a6. Phages were analyzed using the PHASTEST (Wishart et al., 2023) web tool.

#### 2.5.5 Variant calling and within-lineage distances

ONT long reads from *K. pneumoniae* ST307 and ST5994 isolates were mapped to the best, most deeply sequenced assembly from each ST (*i.e.*, samples #9381 and #0868, respectively), using minimap (Li, 2021) v.2.22. For variant calling we used SAMtools (Danecek et al., 2021) mpileup v1.16.1 and iVar (Grubaugh et al., 2019) variants v.1.4. We used only SNP differences and InDels involving 30 or more base pairs, supported by 10 or more reads, with allele frequency > 0.95, 1kbp away from the next variant. Also, we only considered the variation among chromosomes, excluding MGEs and repetitive sequences (integrases, transposons, IS elements, RNAs…). Transmission clusters were defined by single-linkage clustering of pairwise SNP distances at a threshold of 21 variants, following previous studies (David et al., 2019). These parameters were further assessed splitting the 6 most-deeply sequenced FASTQ files into two independent halves and each half processed as a separate sample, which yielded 0–11 discordant SNPs (median 4) between halves of the same isolate. The same comparison without curation yielded 552–1,647 discordant calls, so distances below 4 SNPs are not interpreted here as differences between isolates. Cluster assignment robustness was assessed across a range of thresholds and cross-checked against cgMLST allelic distances.

#### 2.5.6 Phylogenetic análisis

Two maximum-likelihood phylogenies were inferred. The first was designed to test the position of *K. pneumoniae* ST5994 relative to *K. pneumoniae* ST307 and comprised 274 genomes: 63 of the 64 isolates of this study, 150 ST307 representatives from RefSeq, all 51 single- and double-locus variants of ST307 present in RefSeq, all 8 publicly available ST5994 genomes, and two genomes of ST15 and ST258 included to polarize the tree. Core-genome variants were called with Snippy v4.6.0 against the closed ST307 chromosome of isolate #9381 and concatenated with snippy-core. The tree was inferred with IQ-TREE v2.4.0 (Minh et al., 2020) under the model selected by ModelFinder (Kalyaanamoorthy et al., 2017)(TVM+F+ASC+R3) with 1,000 ultrafast bootstrap and 1,000 SH-aLRT (Guindon et al., 2010) replicates. Three topologies were then compared with the approximately unbiased test (Shimodaira, 2002) with 10,000 RELL replicates: the unconstrained maximum-likelihood tree; a topology in which the two sequence types were constrained to reciprocal monophyly; and a topology in which all ST5994 genomes were constrained to a clade together with the Cantabrian ST307 isolates and excluding the 150 ST307 representatives, that is, the hypothesis that ST5994 arose from within the local ST307 population.

A second, larger phylogeny placed the two lineages in the context of the species. All 31,726 *K. pneumoniae* RefSeq assemblies with a complete MLST profile were sketched with Mash (Ondov et al., 2016) (s = 5,000) and compared with the two lineage reference chromosomes, #9381 (ST307) and #0868 (ST5994), separated by D = 0.00641. Genomes falling inside either lineage were removed with a symmetry criterion and the rest ranked by distance to the common ancestor of the pair, dA = (d_307_ + d_5994_ − D)/2, retaining 3 genomes per ST so that the set would avoid over-represent well-sequenced ones. Finally, 393 genomes of 255 sequence types plus all 51 single- and double-locus (SLV and DLV) variants of ST307 in RefSeq, 150 ST307 representatives, all 8 public ST5994 genomes, the 64 isolates of this study and two genomes to polarize the tree gave 668 genomes of 269 sequence types. Core-genome variants were called with Snippy v4.6.0 against the reference chromosome of #9381 (ST307) and concatenated with snippy-core. A maximum-likelihood tree was inferred with IQ-TREE v2.4.0 under the model selected by ModelFinder (GTR+F+ASC+R5), with 1,000 bootstrap and 1,000 SH-aLRT replicates. The tree is rooted on the ST15 genome.

Third, our set of assemblies was compared to all “complete” *K. pneumoniae* genome sequences from NCBI RefSeq (Goldfarb et al., 2025). For this purpose, 1908 K*. pneumoniae* whole genome sequences were downloaded using NCBI datasets (O’Leary et al., 2024) command line tool. These sequences correspond to all genomes tagged as “complete” and uploaded since January 1^st^ 2017 until March 2025. The complete set of 1908 RefSeq assemblies was analyzed using ChewBACCA v.3.3.9 (Silva et al., 2018) to extract a wgMLST schema including 557979 alleles for 15504 loci, after removing 582 paralogs. A 95% cgMSLT was used to assign alleles to our samples and GrapeTree’s MSTree v.2 (Zhou et al., 2018) was used to build up and plot the minimum spanning tree placing our samples’ genomes among those 1908 referenced above.

#### 2.5.7 Chromosomal rearrangements

Structural differences between the two lineage reference chromosomes (#9381 and #0868) were resolved by aligning them with minimap2 v2.22 under the asm5 preset. Alignment blocks of at least 5 kb were taken from the PAF output and classified by strand. Both assemblies begin at the replication origin, (*dnaA*) in each, so neither chromosome had to be rotated before the comparison. Collinear and inverted blocks were plotted from the same alignments. The repeated sequence found at the breakpoints was then searched against both complete reference genomes with BLASTn. The two breakpoint copies were also aligned to each other, with 500 bp of flanking sequence on each side.

#### 2.5.8 Pangenome

The pangenome was built using anvi’o (Eren et al., 2020; Kiefl et al., 2023) suite v.7.2 from 24 K*. pneumoniae* ST307 and 12 ST5994 genomes from this study, together with the published ST5994 genome ARLG-7903 (BioSample SAMN19016674), for a total of 37 genomes. Genomes were downsampled because both lineages contain sets of near-identical genomes that contribute no distinct gene content. To check if this led to artifacts of unequal sampling, the pangenome was recomputed with panaroo v1.8.0 (Tonkin-Hill et al., 2020) in strict clean mode from the Bakta annotations of all 64 genomes (33 ST307, 31 ST5994), and clusters present in one lineage and absent from the other were enumerated at full sampling depth. To quantify the effect of sampling depth itself, the same enumeration was repeated on 200 random subsamples at each of four designs (12 versus 24, 12 versus 12, 24 versus 24 and 31 versus 31), reported in **Supplementary Table 8**.

#### 2.5.9 Statistics

Gene presence/absence was compared between lineages with two-sided Fisher’s exact tests. Genes present in all 64 genomes carry no information and were excluded, the *fosA* locus, present in all 64 and differing only in allele, was tested only once as an allele, leaving 11 genes tested. p values were Benjamini–Hochberg corrected and reported as q values; effect sizes are given as the difference in proportions and as the odds ratio with the Haldane–Anscombe correction. Significance was set at q < 0.05.

### 2.6 Fourier-transform infrared spectroscopy. Development of a machine-learning typing of high-risk clones in KP-CP

A selection of 92 K*. pneumoniae* isolates belonging to the most common ST of CP-KP in our region during the study period were used to perform an automated classification model by using the IR Biotyper (Bruker Daltonics, Bremen, Germany) to predict the main ST of CP-KP. Specifically, we included the following ST and numbers: *K. pneumoniae* ST11 (*n* = 20), *K. pneumoniae* ST5000 (*n* = 17), *K. pneumoniae* ST307 (*n* = 20) and *K. pneumoniae* ST5994 (*n* = 20). Additionally, we included the hypervirulent *K. pneumoniae* (hvKp) clone ST23 (*n* = 15).

Spectra acquisition of bacterial isolates (four technical replicates) was performed following IR Biotyper recommendations and protocols described previously (Vogt et al., 2019), using standardized culture conditions, identical sample preparation procedures and the same acquisition protocol for all isolates. Instrument calibration and spectral quality control were performed before each acquisition run according to the IR Biotyper workflow, ensuring that spectra met the manufacturer’s quality criteria prior to inclusion in the analysis. Spectra were processed by OPUS software V.8.2.28 (Bruker Daltonics, Bremen, Germany) with the corresponding IR Biotyper^®^ software V4.0 (Bruker Daltonics) for data analysis. Data from the area corresponding to polysaccharides (1300–800 cm^-1^) were used for the analysis of differences between isolates. Spectral preprocessing, feature extraction and classifier optimization were performed automatically by the IR Biotyper^®^ software according to the manufacturer’s standardized workflow and were therefore applied uniformly to all spectra. Firstly, the spectra generated were used to construct a dendrogram using Euclidean distance as the exploration method with the UPGMA linkage clustering method to classify the strains according to their ST. Then, to develop an overall classifier for the 5 ST included, a training group and a validation group were set up. The training set was composed of 65 isolates (240 spectra) and the validation set of 27 (106 spectra), following a 70%/30% ratio for each ST in each set, to improve model performance (Xu and Goodacre, 2018). Then, Artificial neural network (ANN) and support vector machine (SVM) algorithms, available in the IR Biotyper software, were used to build the CP-KP classifier, training this process over multiple cycles (300 cycles), and generating a confusion matrix to evaluate the accuracy of the resulting classification model on the training set. The accuracy and recovery of each of the ST was evaluated separately. The performance of the classifier was then evaluated in the validation set. Accuracy was defined as the number of spectra correctly classified (green and yellow results in IR Biotyper). Finally, the classifier was tested with a set of 26 isolates belonging to different STs from those included in the classifier.

## 3 Results

### 3.1 Epidemiology of carbapenemase-producing *Klebsiella pneumoniae*

From January-2021 to December-2024, 448 non-duplicate CP-KP isolates, previously screened by PCR for the detection of acquired carbapenemase genes, were whole-genome sequenced to enable detailed molecular characterization. Most of them (437/448, 97.5%) were *bla*_OXA-48_ producers, 5 isolates (5/448, 1.1%) were *bla*_GES-6_ producers, 3 isolates (3/448, 0.7%) carried a *bla*_KPC-3_, 2 isolates a *bla*_KPC-2_ (2/448, 0.5%) and one isolate (1/448, 0.2%) presented an *bla*_NDM-1_ gene.

Among the CP-KP, 63.6% were identified in infections and the rest were identified in colonization studies (rectal/pharyngeal swabs). The distribution of CP-KP by care unit was as follows: hospital (141/448, 31.5%), intensive-care unit (112/448, 25.0%), primary care (72/448, 16.1%), emergency department (71/448, 15.8%), socio-health center (28/448, 6.3%), consultation (23/448, 5.1%) and unknown (1/448, 0.2%).

In total, 37 different STs were identified in the CP-KP population. Quarterly ST proportion of CP-KP between 2021–2024 is shown in **Figure 1**. Overall, *K. pneumoniae* HRC ST11/OXA-48 was the most common group (48.0%), followed by *K. pneumoniae* ST5000/OXA-48 (15.8%). *K. pneumoniae* ST307/OXA-48 appeared in the 2^nd^ quarter of 2022, becoming the main ST in the 2^nd^ quarter of 2023.

**Figure 1:**
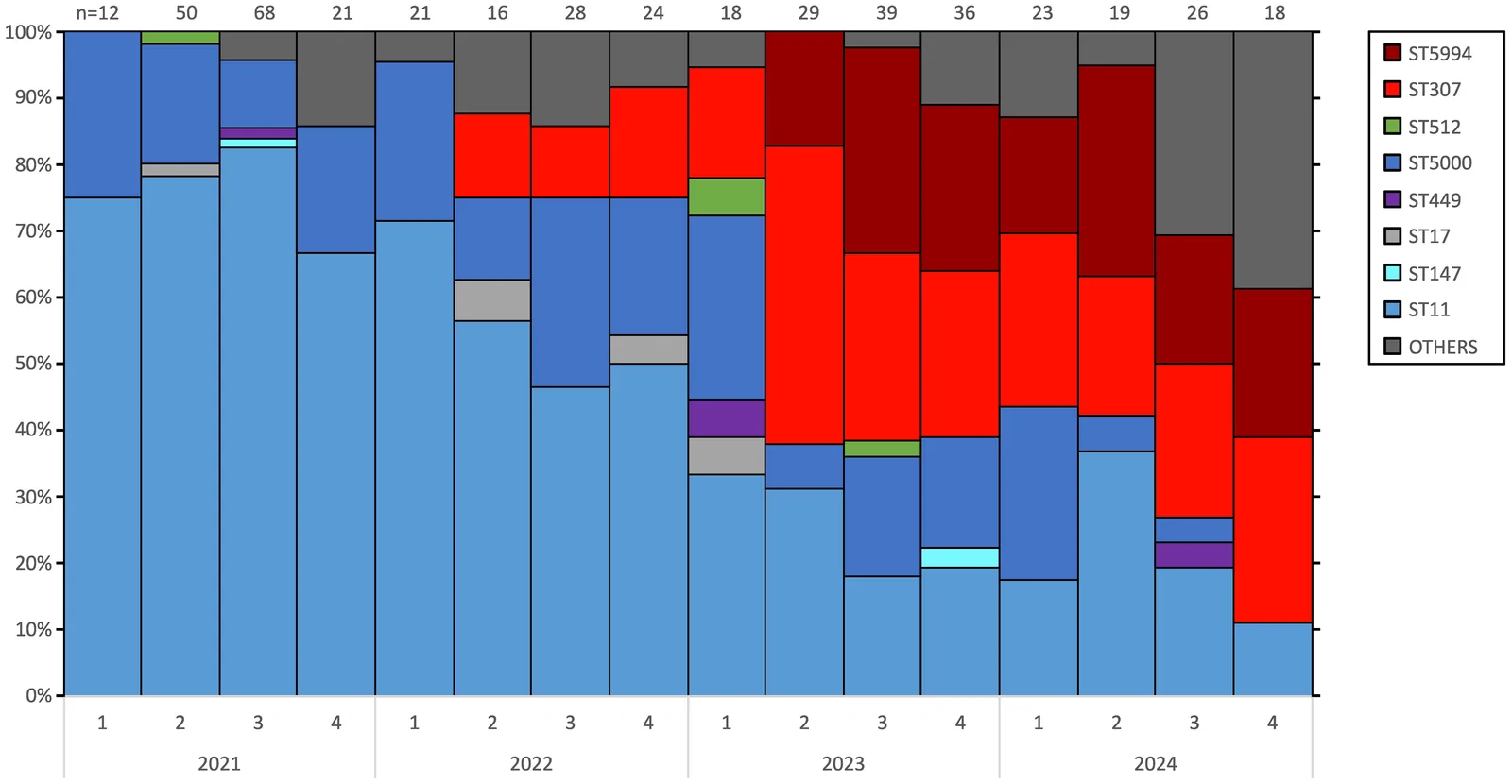
Quarterly ST proportion of CP-KP between 2021–2024 in Cantabria region (Spain). The total number of carbapenemase-producing *Klebsiella pneumoniae* cases in each quarter is shown at the top; Deep and light blue, red, maroon, purple, light grey and green colors indicate most prevalent STs (ST11, ST147, ST307, ST5994, ST449, ST17 and ST512, respectively), while dark grey indicates CP-KPs belonging to other minor STs.

The first detection of *K. pneumoniae* ST5994/OXA-48 happened in the 2^nd^ quarter of 2023, becoming the main ST in the 3^rd^ quarter of 2023. Subsequently, the persistence of clone *K. pneumoniae* ST5994 is confirmed during the year 2024, in which it produced 22.1% of the CP-KP cases, only behind *K. pneumoniae* ST307 (24.4%). The remaining STs were represented by 1–4 isolates.

### 3.2 Emergence of *K. pneumoniae* ST5994 isolates

Since the emergence of clone *K. pneumoniae* ST5994 in our region in May 2023 until December 2024, 45 patients were identified as infected/colonized by CP-KP ST5994. The first 32 cases (until June 2024) were used in this epidemiological and molecular study. *K. pneumoniae* ST5994 isolates were collected mostly from urine (12/32, 37.5%), followed by colonization studies (8/32, 25.0%), blood (3/32, 9.4%) and other locations (9/32, 28.1%). Chronology of appearance and hospital length of stay of patients is shown in **Figure 2A**. In relation to acquisition sites, all cases were healthcare-associated (100%) with 31.3% (10/32) categorized as community onset healthcare-associated (CO-HCA), following previous definitions (Friedman et al., 2002). Most of the patients (81.3%, 26/32) were admitted to the reference hospital (considering both those detected during admission and those who had been hospitalized for two or more days within the previous 90 days), while the 15.6% (5/32) were admitted in long-term care facilities and a single case 3.1% (1/32) was detected in a secondary hospital.

**Figure 2:**
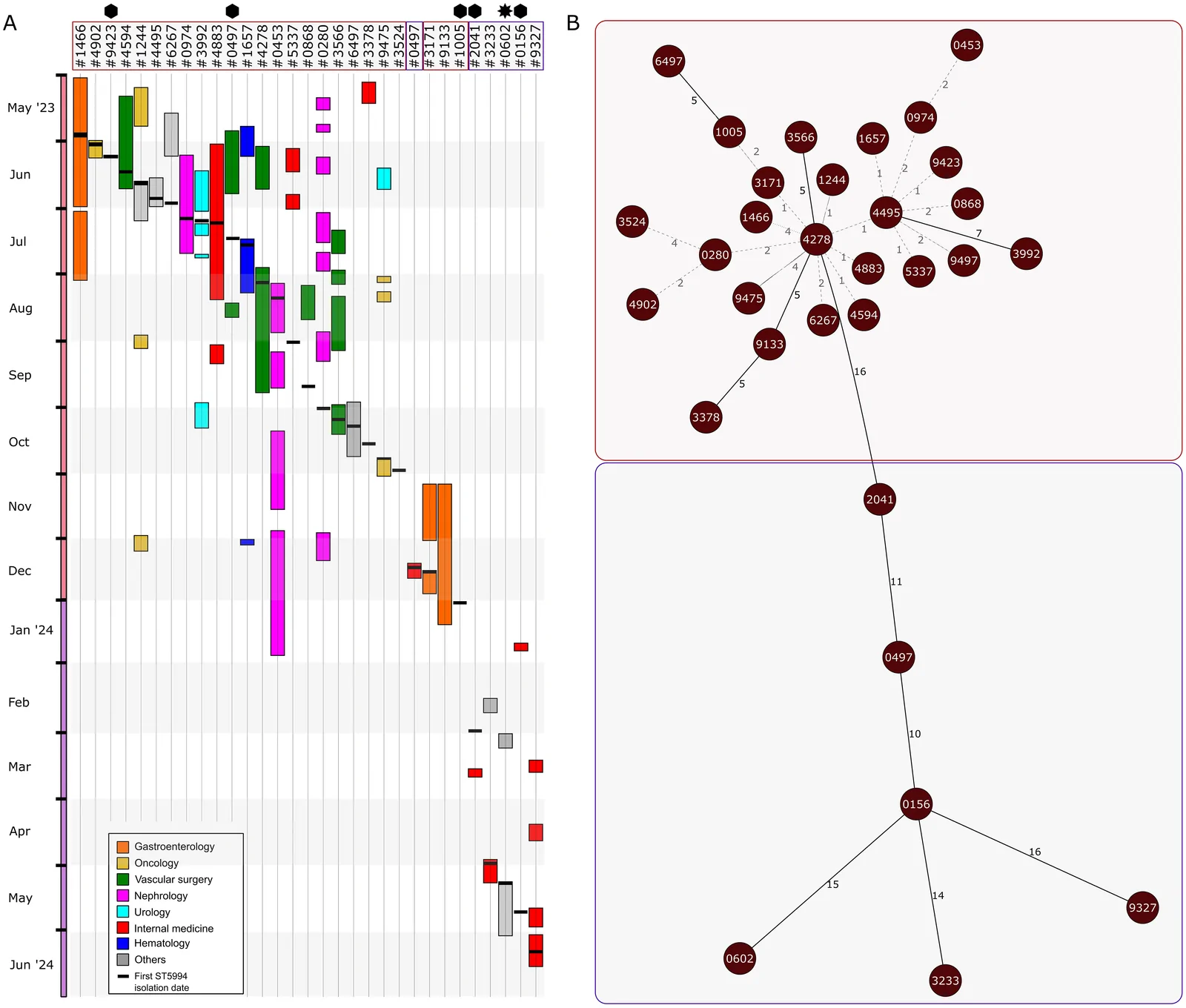
**(A)** Timelines of patients infected/colonized with *K. pneumoniae* ST5994. Patients are sorted according to dates of *K. pneumoniae* ST5994 isolation, from left to right. Medical departments are indicated by square colors (in grey, non-repetitive departments). Sample names are underlined in grey. Hexagons above sample names indicate patients residing in long-term care facilities, eight-pointed star indicates a patient detected in the secondary hospital. **(B)** Minimum spanning tree of core-genome SNP distances among the 31 sequenced *K. pneumoniae* ST5994 isolates. Edge labels give the number of SNPs, with edge length proportional to that number starting from 4 SNPs (pipeline sensitivity floor, below that number edges are dotted). As in any minimum spanning tree, edges of equal length are interchangeable, so the topology should not be read as a genealogy. All 31 isolates form a single cluster at the 21-SNP single-linkage threshold used throughout. For visualization purposes, the longest edge (16 SNPs) and a diverged arm of six isolates are separately highlighted (#0156, #0497, #0602, #2041, #3233 and #9327, blue border), all collected after January 2024.

Considering only cases detected in the reference hospital (n=26), inpatients with *K. pneumoniae* ST5994 were admitted to 14 different medical departments. Examining the chronology of the appearance of patients identified with ST5994 (**Figure 2A**) shows that, in most cases, they did not coincide in the same medical department. And in cases where there is spatial overlap, the patients were not admitted at the same time. Therefore, there is no apparent close contact between the patients. These findings do not support a single outbreak in one hospital department, as most patients did not overlap in time and place during their hospital stay. Instead, the observed epidemiological pattern is compatible with sustained dissemination across multiple hospital wards, although the underlying transmission routes could not be determined with the available data. Conversely, a direct chain of transmission may have occurred in the vascular surgery department, where at least 3 patients have temporal overlaps during the admission period in July 2023.

We found only 2 K*. pneumoniae* ST5994 sequences in the *Klebsiella* Pasteur MLST database (bigsdb.pasteur.fr, accessed in October 2025) and one more in NCBI, deposited as BioSamples SAMN19016674, SAMN50739038 and SAMEA117985995, coming from multi-isolate projects (Faccone et al., 2023) in USA, Argentina and Colombia (Medina et al., 2025), respectively. We found no additional examples of *K. pneumoniae* ST5994 among 1908 complete genomes in NCBI datasets uploaded between 2017 until March 2025. Typing all 31,726 *K. pneumoniae* genomes available in RefSeq (consulted in July 2026) by allelic profile, rather than restricting the search to closed genomes, identified six public ST5994 assemblies: three from Mexico (2021), two from Argentina (2021 and 2023) and one from the Dominican Republic (2018). Two further ST5994 genomes are deposited in GenBank only, from the USA and Colombia, and are invisible to a RefSeq-derived search by construction, giving eight public genomes in total (**Supplementary Table 7**). Seven of the eight are draft assemblies and would not be retrieved by a search restricted to complete genomes. None of the eight carries a *bla*_OXA-48_ gene: two carry *bla*_NDM-5_, one *bla*_KPC-_3, one *bla*_KPC-3_, and four carry no carbapenemase gene. The isolates reported here therefore represent, to our knowledge, both the first description of ST5994 in Europe and the first association of this sequence type with OXA-48 anywhere. The earliest public genome of the lineage, and the only one of environmental origin, was recovered from the Ozama River (Dominican Republic) in 2018, so ST5994 has been present in the Americas since at least 2018 in both clinical and environmental settings. For comparison, 1,233 RefSeq genomes carry the exact ST307 allelic profile.

### 3.3 Within-lineage genomic diversity and transmission structure

Whole genome sequences of 33 K*. pneumoniae* ST307 and 31 K*. pneumoniae* ST5994 were used to compare their genomes and establish molecular phylogenies. Three alleles change between *K. pneumoniae* ST307 and *K. pneumoniae* ST5994: *pgi* (52 ->37), *phoE* (1->21) and *tonB* (7->64). We tracked chromosome variation of the 31 K*. pneumoniae* ST5994 as described above.

After curation, the median pairwise distance was 5 SNPs among the 33 ST307 isolates (range 0–36, 66 variable sites) and 8 SNPs among the 31 ST5994 isolates (range 1–36, 89 variable sites). At the 21-SNP single-linkage threshold each lineage formed a single cluster containing all of its isolates, and this assignment was stable across the range of thresholds examined (**Supplementary Table 5**). The two lineages nonetheless differed markedly in the structure underlying that identical cluster count. Fifteen ST307 genomes were indistinguishable from one another (0 SNPs), together with two further pairs of identical genomes, so that the 33 isolates collapsed to 17 distinct genotypes (**Supplementary Figure 1**); among the ST5994 isolates no two genomes were identical and all 31 represented distinct genotypes (**Figure 2B** and **Supplementary Table 6**). Accordingly, the ST307 network took the form of a star centered on a single dominant genotype, whereas the ST5994 network formed a chain with a median step of 2 SNPs between neighboring genotypes and a diverged arm of six isolates (#0156, #0497, #0602, #2041, #3233 and #9327) joined to the remainder through a single 16-SNP link. This group includes the only patient detected in the secondary hospital and three of the five patients admitted to long-term care facilities, each from a different center. The greater median distance within ST5994 therefore reflects diversity distributed along a transmission chain rather than greater divergence between individual isolates. All six isolates of the diverged arm were collected after January 2024.

### 3.4 Phylogenetic position of *K. pneumoniae* ST5994 relative to ST307

To determine whether *K. pneumoniae* ST5994 arose from within the ST307 population or represents a separate lineage, a maximum-likelihood phylogeny was inferred from 274 genomes comprising ST307 genomes, SLV/DLVs, ST5994 and close relatives, with two outgroup genomes (TVM+F+ASC+R3). ST307 and ST5994 were recovered as reciprocally monophyletic sister clades with maximal support (SH-aLRT 100%, ultrafast bootstrap 100%), with the root falling outside both. The minimal clade containing all 39 ST5994 genomes contained those 39 and no other genome, and the minimal clade containing the 182 ST307 genomes contained 216 tips — the 182 ST307 genomes and 34 SLV/DLV variants — and no ST5994 genome. The nested topology was rejected (p_AU_ = 4.9 × 10^−4^; 663 log-likelihood units worse), whereas the first two could not be distinguished (p_AU_ = 0.32 and 0.68*). K. pneumoniae* ST5994 is therefore a lineage related to, but not derived from, ST307.

The analysis was repeated on 668 genomes spanning 269 sequence types (GTR+F+ASC+R5; 195,805 variable sites, 120,689 parsimony-informative). The topology was fully reproduced (see detail in **Figure 3A** and complete tree in **Supplementary Figure 6**), the clade containing all 39 ST5994 genomes contained no other genome; the clade containing all 183 ST307 genomes contained 218 tips, (183 plus 35 SLV/DLVs of ST307), and no ST5994 genome. The nearest lineage outside the pair was ST587 (ten genomes), followed by two ST6337 genomes falling on the ST307 side. *K. pneumoniae* ST307 is thus paraphyletic with respect to its own single-locus variants, but not with respect to ST5994. We note that no genome branches between ST5994 and the common ancestor of the two lineages, whereas 36 genomes of ten lineages branch on the ST307 side. As this reflects what has been sequenced and deposited, it is reported as an observation about the available data rather than about the lineages themselves.

**Figure 3:**
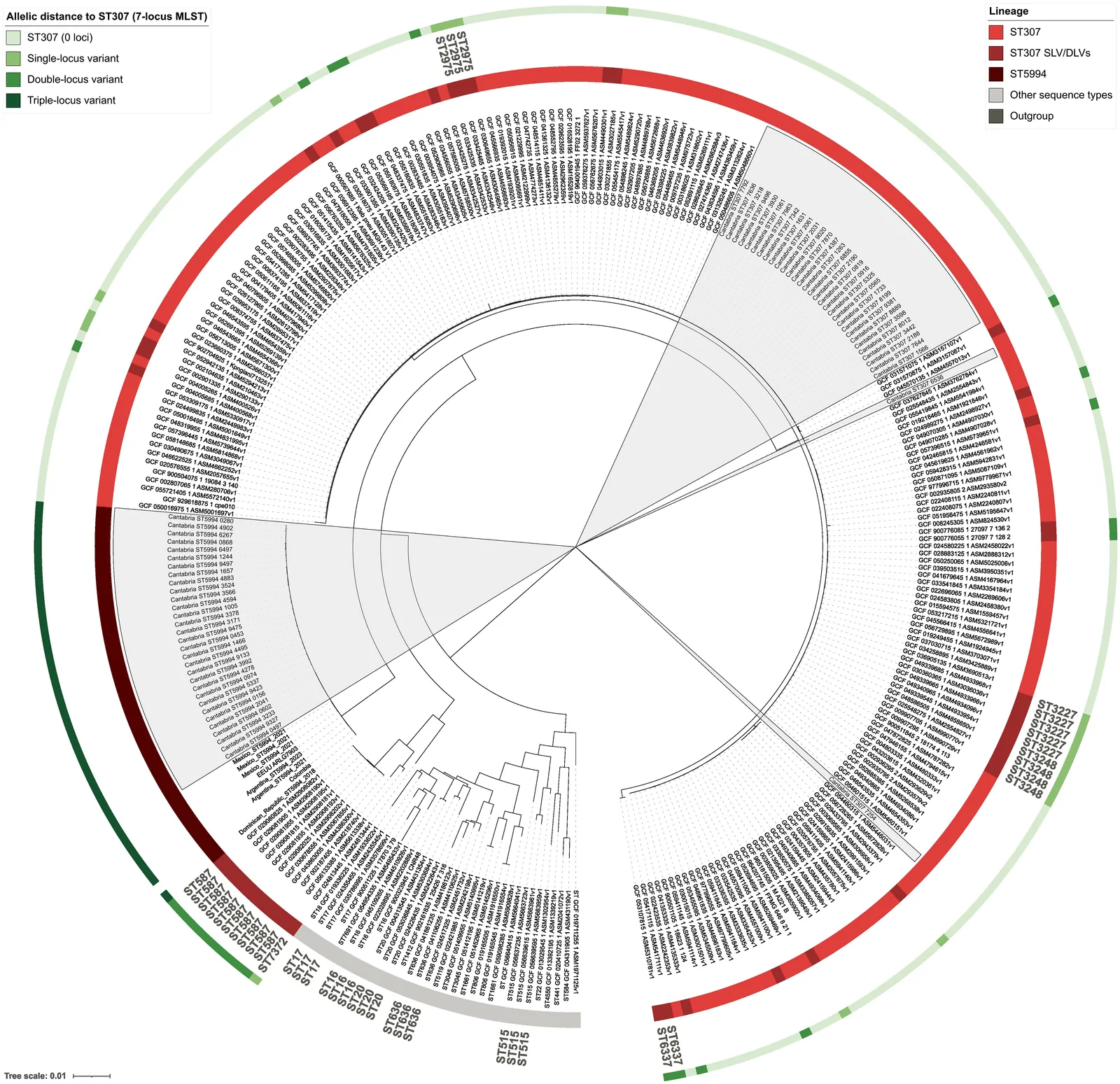
Maximum-likelihood phylogeny of 300 genomes spanning 25 sequence types (detail). Pruned tree of 300 leaves from a major (see **Supplementary Figure 7**) maximum-likelihood phylogeny of 668 genomes spanning 269 sequence types, from 195,805 core-genome variable sites under GTR+F+ASC+R5. Radial grey areas indicate the sequences from this study. Inner ring: Lineages as related to either ST 307 (light red), single- or double-locus variants of ST307 (dark red), ST5994 (maroon) and other close sequence types (grey). Medium ring: Sequence types of some closely related clades, either inside ST307 (ST3227, ST3248, ST6337, ST2975) or outside both ST307 and ST5994 (ST587, ST7372). Outer ring: Number of locus variants with respect to ST307 according to the 7-loci schema.

We built a pangenome using 1908 K*. pneumoniae* whole-genome sequences available in NCBI datasets, along with the 64 genomes from our study. Based on their pangenome, we constructed a minimum-spanning tree (MS-Tree, **Figure 3**). The *K. pneumoniae* ST307 genomes from our study (highlighted yellow) formed a differentiated phylogenetic cluster among the 1908 publicly available *K. pneumoniae* genomes and within a broader branch with *K. pneumoniae* ST307 genomes (see **Supplementary Figures 7**, **8**).

### 3.5 Chromosomal architecture and accessory genome content

By comparing the chromosome sequences of the representative genomes [samples #0868 (ST5994) and #9381 (ST307)], we found a large chromosomal rearrangement between the two reference sequences (**Figure 4A**), involving a single inversion falling between positions 1,844,108 and 4,008,270 of the *K. pneumoniae* ST307 chromosome, an interval of 2.16 Mbp (40% of total length), with some extra prophage and transposon insertions and deletions. The inverted segment is centered at position 2,926,189, that is, 217,522 bp from the estimated *terminus*, approximately symmetrical about the replication terminus, preserving the balance between the two replichores, which is the configuration under which large chromosomal inversions are tolerated in Enterobacteriaceae (Eisen et al., 2000). Both breakpoints in the *K. pneumoniae* ST5994 chromosome are flanked, immediately outside the inverted segment, by a copy of the same mobile element in opposite orientations: a group II intron encoding a reverse transcriptase/maturase. The two copies are 99.7% identical over 1,934 bp, the homology extending approximately 380 bp beyond the 1,485 bp coding sequence. The loci shared by both lineages surrounding these sites (genes *lexA, dinP, lysR, fabG, araJ, dinI, rfaF, araC* and *cynX*), occur in mirrored synteny in the two reference genomes. The element occurs in seven copies in the ST5994 reference (four chromosomal) and three in the ST307 reference (one chromosomal), all 3 shared with ST5994, which have 4 specific copies, two of them flanking the inversion. Thus, the element has proliferated within the ST5994 lineage, and the inversion post-dates that proliferation. Homologous recombination between inversely oriented copies of a dispersed repeat is a well-described route to large chromosomal inversions in bacteria (Achaz et al., 2003). Insertion post-inversion cannot be ruled out, but recombination between pre-existing copies requires no additional assumption, whereas the alternative requires two independent insertions landing at both junctions in the appropriate orientations. We therefore report this as the most parsimonious mechanism, not demonstrated, consistent with the data. The same comparison against the only closed public ST5994 genome (GCF_052373055.2, Argentina) recovered the ST307 arrangement, so the inversion characterizes the Cantabrian ST5994 population rather than the ST5994 lineage.

**Figure 4:**
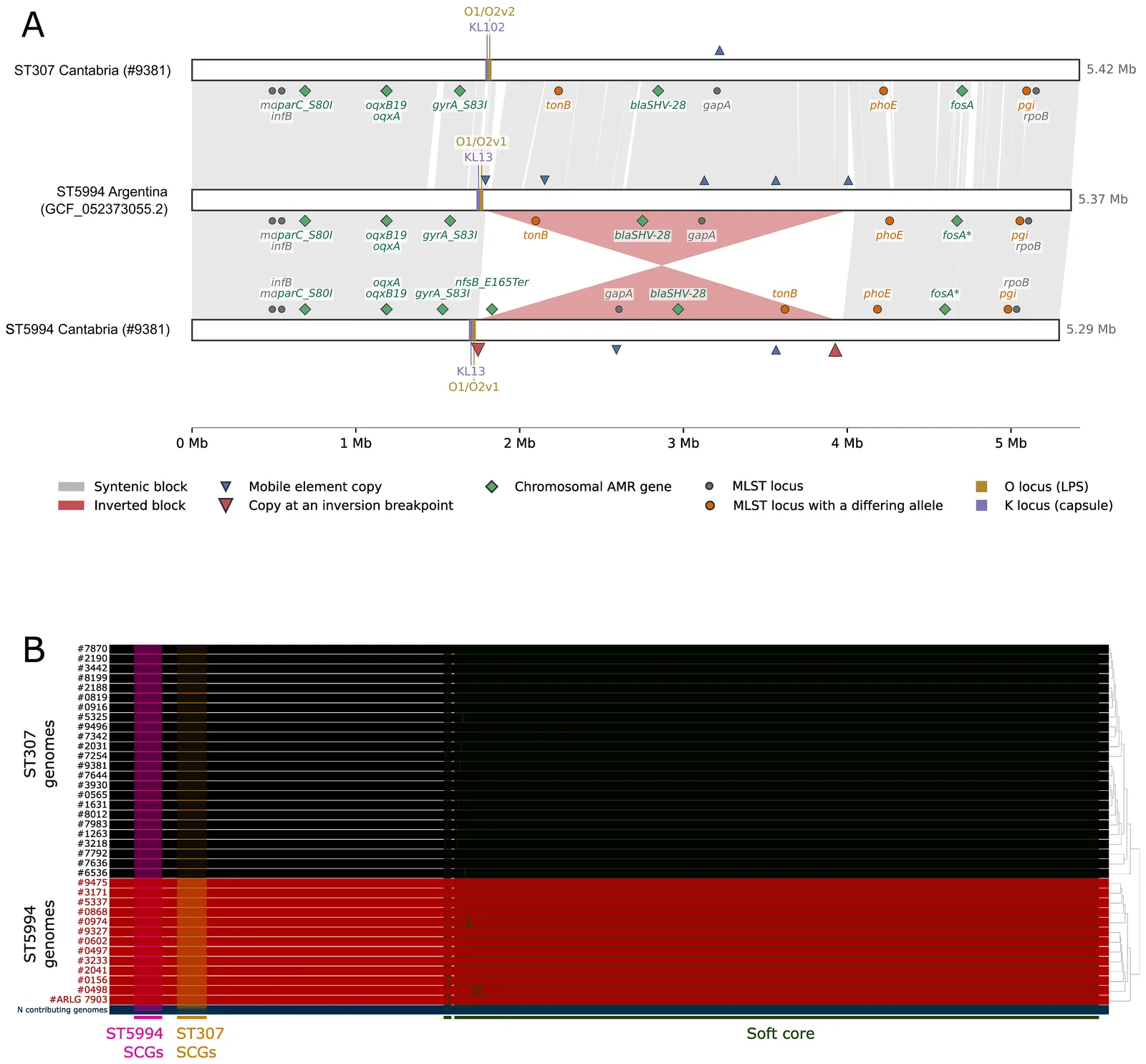
A single large chromosomal inversion distinguishes the Cantabrian ST5994 population from both ST307 and the only closed public ST5994 genome. **(A)** Chromosome-scale synteny among *K. pneumoniae* ST307 (#9381), the closed public ST5994 genome from Argentina (GCF_052373055.2) and the Cantabrian ST5994 reference (#0868), all oriented to a common origin at *dnaA*. Gray ribbons denote syntenic and red crossed ribbons inverted blocks. Triangles mark chromosomal copies of the 1,485 bp mobile intronic element, pointing up or down according to strand, red ones marking the two copies at the inversion breakpoints. **(B)** *K. pneumoniae* ST307 (black, top, n=24) and *K. pneumoniae* ST5994 (red, bottom, n=12, plus ST5994 Argentinian ARLG-7903 genome from NCBI) pangenome alignment of selected samples (see left axis). Single Cluster Genes (SCGs) are depicted in horizontal axis, with those shared by >95% genomes labelled as “soft core” at right, those shared by a subset at left and those SCGs that are unique to a sample in the middle. SCGs belonging to only either *K. pneumoniae* ST5994 or *K. pneumoniae* ST307 samples are underlined in pink and orange, respectively. Right, dendrogram of presence-absence of SCGs.

The inversion is supported by long reads spanning both breakpoints in all 31 Cantabrian ST5994 isolates (3–236 reads per breakpoint, median 43) and in seven further ST5994 read sets from the same collection, whereas the ST5994 reference yields no read spanning either breakpoint of the ST307 arrangement at 377× and 120× depth.

Across all 64 genomes the pangenome comprised 7,389 gene clusters, 4,529 of them present in every genome. At full sampling depth, 436 clusters were present in one or more *K. pneumoniae* ST5994 genomes and absent from all ST307 genomes, and 1,592 the reverse. This asymmetry is driven by sporadic rather than lineage-level content: 1,047 of the 1,592 ST307-specific clusters (66%) occurred in fewer than 10% of ST307 genomes, against 119 of 436 (27%) for ST5994. When restricted to clusters fixed within their lineage (≥95% of genomes) the two are comparable, 228 for ST5994 and 261 for ST307. *K. pneumoniae* ST5994 therefore carries substantially less accessory content, while both lineages carry similar numbers of fixed lineage-specific clusters.

Capsule and O-locus types were invariant within each lineage and are shared with the public ST5994 genomes, indicating that both are lineage-level characters of the Cantabrian expansion. The single exception is the ST5994 genome from Argentina (GCF_052373055.2), in which a 2,165 bp deletion truncates two CDS of the O-locus and switches the predicted type from O1ab to O2a. The deleted segment is replaced in that genome by a 763 bp IS1-family transposase. We then searched for IS*1* copies within the O locus in every genome in which the locus could be delimited by both flanking anchors, namely the 31 ST5994 isolates of this study and six of the eight public ST5994 assemblies, the remaining two being drafts in which the locus is split across contigs. A similar copy of IS1 inside the O locus was not found in any other genome. The element was nonetheless present elsewhere in all 31 isolates of this study, at 5 to 9 copies per genome, the nearest of them at 649 kb from the locus. The association between the deletion and the IS*1* insertion is therefore specific to that genome rather than a general feature of the lineage.

Among the clusters fixed in one lineage and absent from the other, four genes of the KL13 capsule locus (including *wcaJ*) were specific to ST5994 and five of the KL102 locus (including *wza* and *wbaP*) to ST307, consistent with the capsule types assigned independently by *Kleborate*. Nine clusters encoding a type VI secretion system (*tssB, tssF, tssJ, tssK, tssL, impA, vgrG, vasK/imcF* and *dotU*) were present in at least 31 of the 33 ST307 genomes and in none of the 31 ST5994 genomes, as were clusters for citrate fermentation, sorbose utilization and a cell-wall polysaccharide biosynthesis operon.

### 3.6 Antimicrobial susceptibility testing and antimicrobial resistance genes

Antimicrobial susceptibility testing was performed on 33 K*. pneumoniae* ST307 and 32 K*. pneumoniae* ST5994 isolates (**Table 1**). Ceftazidime/avibactam, colistin and amikacin were the most active antimicrobials, each showing 100% susceptibility among both *K. pneumoniae* ST307 and *K. pneumoniae* ST5994 isolates. Imipenem/relebactam and meropenem/vaborbactam demonstrated >93.9% susceptibility across the entire collection. *K. pneumoniae* ST5994 shows comparable susceptibility rates to *K. pneumoniae* ST307. The largest differences were observed for imipenem (93.8% vs. 78.1%), gentamicin (87.5% vs. 6.1%) and fosfomycin (75.0% vs. 0.0%), with *K. pneumoniae* ST5994 isolates being more susceptible.

**Table 1:** Antimicrobial susceptibility of *K. pneumoniae* ST307 and *K. pneumoniae* ST5994 isolates.

	*K. pneumoniae* ST307 (*n* = 33)				*K. pneumoniae* ST5994 (*n* = 32)			
Antibiotic	S (%)^a^	R (%)^a^	MIC_50_^b^	MIC_90_^b^	S (%)^a^	R (%)^a^	MIC_50_^b^	MIC_90_^b^
Amoxicillin/clavulanate	0.0	100	≥64	≥64	0.0	100	≥64	≥64
Piperacillin/tazobactam	0.0	100	≥128	≥128	0.0	100	≥128	≥128
Cefotaxime	0.0	100	≥64	≥64	0.0	100	≥64	≥64
Ceftazidime	3.3	96.7	32	≥64	0.0	100	32	≥64
Ceftazidime/avibactam	100	0.0	≤0.25	0.5	100	0.0	0.5	0.5
Ceftolozane/tazobactam	6.3	93.7	≥8	≥8	6.3	93.7	≥8	≥8
Aztreonam	7.1	92.9	16	≥64	0.0	100	32	≥64
Ertapenem	15.2	84.8	2	≥8	0.0	100	≥8	≥8
Imipenem	78.1	18.7	≤1	≥8	93.8	3.1	≤1	2
Imipenem/relebactam	94.4	5.6	1	≥8	95.5	4.5	0.5	2
Meropenem	84.8	9.1	1	8	78.1	3.1	1	4
Meropenem/vaborbactam	93.9	6.1	≤0.5	4	96.4	3.6	≤0.5	2
Amikacin	100	0.0	4	4	100	0.0	4	4
Gentamicin	6.1	93.9	≥16	≥16	87.5	12.5	≤1	≥16
Tobramycin	7.7	92.3	8	≥16	0.0	100	8	≥16
Ciprofloxacin	3.0	97.0	≥4	≥4	0.0	100	≥4	≥4
Trimethoprim/sulfamethoxazole	3.0	97.0	≥320	≥320	6.3	93.7	≥320	≥320
Fosfomycin	0.0	100	≥256	≥256	75.0	25.0	≤16	≥256
Colistin	100	0.0	≤0.5	≤0.5	100	0.0	≤0.5	≤0.5

a S (susceptible, standard dose); R, resistant.

b Expressed in mg/L.

An average of 15.9 genes were detected in *K. pneumoniae* ST307 (range: 13-21) and 15.0 in *K. pneumoniae* ST5994 (range: 14-17) (see **Supplementary Tables 3**, **4**). Three strains (#2188/ST307, #3442/ST307 and #9475/ST5994) were resistant to imipenem, imipenem/relebactam, meropenem and meropenem/vaborbactam. Analysis of porin genes (*ompK35* and *ompK36)* revealed that isolate #2188/ST307 presented a truncated *ompK35* gene lacking the 5’ region up to nucleotide position 384 (where an ATG start codon is located), while in #3442/ST307, *ompk36* is not present. Both lineages carried a chromosomally encoded a 139-aa FosA5-family fosfomycin-modifying enzyme (Guo et al., 2016), invariant within lineages, at 1–4 SNPs from reference *fosA6* and differing between lineages by one synonymous (codon 7) and two non-synonymous (L25Q, Q139E) mutations. ST307 allele differed from *fosA6* (NG_051497.1) at a single position and the ST5994 allele from *fosA10* (NG_047884.1) at three positions; this new ST5994-specific allele has been deposited in GenBank under accession PZ791900. All other examined fosfomycin susceptibility determinants (GlpT, UhpT, UhpA, CyaA, PtsI, MurA) shared identical sequences between lineages. Conversely, the 1.7-kb upstream region (*lysR–mdaB*) was substantially more divergent (2.8% sequence divergence and homology loss at -1,771 bp), showing lineage-specific synteny differences around *fosA* without changes in gene content.

Eleven genes were present in all 64 genomes (**Supplementary Tables 3**, **4**), together with *fosA*. Comparing the presence of the remaining genes between lineages by Fisher’s exact test with Benjamini–Hochberg correction, only two of the eleven variable genes differed significantly: the *fosA* allele (31/31 ST5994 versus 0/33 ST307, q = 6.2 × 10^−18^) and *aac(3)-IIa* (1/31 versus 31/33, q = 9.8 × 10^−14^, odds ratio 256). All remaining genes returned q = 1.The two lineages therefore share essentially one resistome, and differ in one chromosomal allele and one plasmid-borne acetyltransferase.

### 3.7 Virulome analysis and biofilm production

We searched for virulence-associated genes among CP-KP, including those of the *K. pneumoniae* ST307 and *K. pneumoniae* ST5994 clones. However, only *wabG*, *ureA* and *uge* genes, which are commonly found in the core genome of both classical strains and hvKp (Kot et al., 2025), were consistently found in both groups. Kleborate v3.1.2 assigned a virulence score of 0 to all 31 ST5994 genomes: none carried yersiniabactin, colibactin, aerobactin, salmochelin, *rmpADC* or *rmpA2*. Among the 33 ST307 genomes, 17 carried the yersiniabactin locus *ybt 10* on ICE*Kp4*, although in 16 of these the locus was truncated. Capsule typing was uniform within each lineage, with KL102 in 33 of 33 ST307 genomes and KL13 in 31 of 31 ST5994 genomes; O locus typing gave *O1ab* in all 31 ST5994 genomes and *O2afg* in 32 of 33 ST307 genomes (#1631 was O12). *K. pneumoniae* ST5994 therefore carries less acquired virulence determinants than the lineage it displaced, and its regional expansion is not attributable to acquired virulence genes.

### 3.8 Mobile genetic elements

All *K. pneumoniae* ST307 and *K. pneumoniae* ST5994 isolates carried two plasmids: a large 192 kbp IncFIB(K) plasmid containing multiple AMR genes (**Supplementary Figure 2**) and a smaller, IncL (PTU-L/M) pOXA-48 plasmid. Both plasmids were predicted to be conjugative, the first encoding a MOB_F_ relaxosome and MPF_F_ conjugative pilus, and the second a MOB_P_ relaxase and MPF_I_ pilus. The IncFIB(K) plasmid harbored ten AMR genes, including *tet*A, *aac*(6’)-Ib-cr, *aac*(3’)-IIe, *aph*(6)-Id, *aph*(3”)Ib, *dfr*A14, *sul*2, *bla*_CTX-M-15_, *bla*_OXA-1_ and *bla*_TEM-1_, and contained two prophages identified by PHASTEST and Phigaro (highlighted in yellow, in **Supplementary Figure 2**). A *catB3* sequence was also detected in all genomes, with a shared truncation at 70% of its total length in all genomes.

The IncF plasmid of ST5994 isolates lacks a segment of about 50 kbp comprising metabolic operons *glg*, *lac* and *fec*. The *fecRABCDE* operon is exclusive to the IncF plasmid, hence rendering the ST5994 strains without this operon. The plasmid pOXA-48 presented only some SNPs in the conjugative region, present in some *K. pneumoniae* ST307 strains, as shown in **Supplementary Figure 3**, with no remarkable consequences in their resistance phenotype.

### 3.9 Rapid detection of *K. pneumoniae* ST5994 using FT-IR Biotyper

A dendrogram (**Supplementary Figure 5**) using all 92 CP-KP isolates was constructed to check the correct ST distribution using the IR Biotyper. The classifier, built from 240 spectra using an ANN algorithm, showed 91-100% precision and 98% accuracy (**Supplementary Table 1**). Then, the classifier was validated with the validation set composed of 106 spectra not included in the training set. Detection rates by ST were as follows: *K. pneumoniae* ST23 (100%, isolate classification score 23.8-100%), *K. pneumoniae* ST5994 (100%, isolate classification score 94-100%), *K. pneumoniae* ST307 (100%, isolate classification score 97-100%), *K. pneumoniae* ST11 (95.5%, isolate classification score 50-100%) and *K. pneumoniae* ST5000 (75%, isolate classification score 0-100%).

Clades were consistent with WGS phylogeny: *K. pneumoniae* ST5994 and *K. pneumoniae* ST307 clustered together, and *K. pneumoniae* ST11 and *K. pneumoniae* ST5000 appear intermixed. None of the isolates belonging to others ST were misclassified as one of the target ST. A list of isolates and their STs included in this test appears in **Supplementary Table 2**.

## 4 Discussion

The epidemiology of carbapenemase-producing *Klebsiella pneumoniae* is characterized by a continuous succession of high-risk clones (Mathers et al., 2015; Peirano et al., 2020). Our results confirm previous surveillance reports in which *bla*_OXA-48_ was the most common carbapenemase in Spain (Cañada-García et al., 2022). However, we found a divergence in clonal dominance in our region, Cantabria, compared to the national trend: while HRC ST307/OXA-48 and ST512/KPC-3 dominated CP-KP epidemiology in Spain from 2021 to 2023 (RedLabRA et al., 2023), *K. pneumoniae* ST11 and its close relative *K. pneumoniae* ST5000 accounted for 93% of cases in Cantabria, in 2021. By the end of this study, their prevalence had dropped to 11% as *K. pneumoniae* ST307 and the newly emerged *K. pneumoniae* ST5994 became dominant (**Figure 1**). Precisely, *K. pneumoniae* ST5000 had recently been described as a new MLST closely related to *K. pneumoniae* ST11 that was first described in our region (Alioto et al., 2023).

The local dissemination of 32 K. pneumoniae ST5994 cases represents a notable epidemiological event: at the time of writing only eight ST5994 genomes were publicly available worldwide, none of them from Europe and none carrying *bla*_OXA-48_. The genomic structure of the expansion is consistent with the epidemiological picture. All 31 sequenced isolates fell within a single 21-SNP cluster, yet no two genomes were identical and the network formed a chain rather than a star, which is the signature of continued transmission with accumulating diversity rather than of a point-source event. The available epidemiological data supports this chain of transmission model, as most patients did not overlap in time or location within the hospital. Previous experience has shown that hidden reservoirs, workers’ hands or contaminated surfaces may sustain nosocomial transmission without apparent contact between patients (García-Fernández et al., 2019; Marimuthu et al., 2022). In addition, patients passing through emergency rooms or diagnostic common areas also contribute to the maintenance of hospital transmission (Murray et al., 2017). Patient mobility likely contributes to the spread of transmission (Huang et al., 2010). Taken together, these observations are compatible with several possible indirect transmission scenarios, including healthcare-associated transmission or unsampled environmental reservoirs. However, given the retrospective nature of this study the definitive transmission routes could not be established.

*K. pneumoniae* ST5994 and *K. pneumoniae* ST307 are sister lineages that differ in capsule locus (KL13 versus KL102), in the chromosomal *fosA* allele and in a chromosomally encoded type VI secretion system, all three of which are shared with the public ST5994 genomes and are therefore properties of the lineage. Superimposed on that background, the Cantabrian population carries a 2.16 Mb chromosomal inversion that the only closed public ST5994 genome does not. Such rearrangements have previously been associated with bacterial adaptation under clinical pressure in other studies (Wedel et al., 2023). Also, the *K. pneumoniae* ST5994 lineage lacks a segment of the IncFIB(K) plasmid, containing operons for lactose, glycogen, and ferric citrate metabolism. This reduction in the accessory genome, combined with the absence of the Type VI secretion system (T6SS) in the chromosome, is consistent with genomic changes previously reported during the evolution of successful clinical lineages, although their biological significance in ST5994 remains to be determined (Zhang et al., 2025).

From a therapeutic standpoint, *K. pneumoniae* ST5994 exhibits a multidrug-resistant profile comparable to *K. pneumoniae* ST307, yet it shows increased susceptibility to specific agents. The difference in gentamicin resistance between ST307 and ST5994 was associated with the presence of the *aac(3)-IIa* gene in 31 out of 33 (93.9%) of the ST307 isolates *versus* only 1 out of 31 ST5994 isolates. The fosfomycin susceptibility gap between ST5994 (75.0%) and ST307 (0.0%) is hardly explained by only two conservative amino-acid differences in the FosA5 enzyme (including a C-terminal Q139E), while every other determinant was identical. The much greater divergence of the upstream region and the abrupt loss of homology point to chromosomal reorganization. Whether it translates into differential *fosA* expression, and then susceptibility difference, requires transcriptional characterization, beyond the scope of this study. The fosfomycin resistance phenotype in 25% of ST5994 isolates indicates that other factors contribute.

The antimicrobial susceptibility results are consistent with previous multicenter studies conducted in Spain, in which ceftazidime/avibactam showed superior activity against OXA-48-producing isolates compared with carbapenems and other new beta-lactam/beta-lactamase inhibitors (Vázquez-Ucha et al., 2021; Cañada-García et al., 2022). However, the emergence of high-level resistance to carbapenems and new β-lactam/β-lactamase inhibitor combinations in specific isolates (*e.g.*, #2188 and #9475) is concerning. This resistance is driven by the truncation or loss of porin genes ompK35 and ompK36, a well-documented mechanism that limits drug entry and synergizes with carbapenemase production (Blanco-Martín et al., 2024).

ST5994 carries a smaller accessory virulome than ST307: no ST5994 genome carries the ICE*Kp4* element or the yersiniabactin (*ybt*) operon. This same absence was also observed in all public available *K. pneumoniae* ST5994 genomes. Data presented by Lam and coauthors showed that different *ybt* and *clb* lineages circulate freely in the *K. pneumoniae* population through ICEKp elements, highlighting the broad dissemination of accessory virulence factors (Lam et al., 2018).

Importantly, both *K. pneumoniae* ST307 and *K. pneumoniae* ST5994 maintained the core machinery for biofilm formation, including the *mrkABCD* (Type 3 fimbriae) and *fimAH* (Type 1 fimbriae) operons. While Type 1 fimbriae play a supportive role, Type 3 fimbriae are crucial for strong biofilm formation (Schroll et al., 2010). Although biofilm formation has been associated with environmental persistence and healthcare-associated dissemination in previous studies (El Naggar et al., 2024), the present study was not designed to evaluate the contribution of biofilm production to the transmission dynamics of ST5994. Therefore, any relationship between the observed biofilm phenotype and the regional dissemination of this lineage should be interpreted with caution.

Regarding their plasmid content, both IncFIB(K) and IncL (PTU-L/M) pOXA-48 plasmids (Poirel et al., 2012; Hamprecht et al., 2019; Fernández-Calvet et al., 2023) had been previously described in other high-risk CP-KP lineages such as ST11, ST101, ST405, and ST307 (Baquero and Coque, 2011; Poirel et al., 2012). These mobile plasmids have broad host ranges, being capable to spread their AMR content to neighboring or coinfecting bacteria (Adamczuk et al., 2015).

Rapid identification of emerging HRCs is critical for infection control. Our study demonstrates that Fourier-Transform Infrared (FT-IR) spectroscopy, coupled with machine learning, can accurately discriminate *K. pneumoniae* ST5994 from *K. pneumoniae* ST307 and other major clones. This discrimination is likely driven by the significant K-locus shift (from KL102 in *K. pneumoniae* ST307 to KL13 in *K. pneumoniae* ST5994). The technique could not fully resolve the closely related *K. pneumoniae* ST11 and *K. pneumoniae* ST5000 strains, possibly due to ST5000 being very closely related to ST11 (Alioto et al., 2023), having very similar polysaccharide region (Hu et al., 2021) (1300–800 cm^-1^). Still, it remains a powerful tool for monitoring the expansion of emerging clones like *K. pneumoniae* ST5994.

Several limitations should be acknowledged. First, although CP-KP surveillance was conducted at the regional level, the emergence of ST5994 was primarily detected in a single tertiary reference hospital, making this essentially a single-center study for this clone. Second, not all ST5994 isolates detected during the study period were included in the comparative genomic analysis, which may have limited the reconstruction of later transmission events. Third, the epidemiological investigation was retrospective and based mainly on admission dates, hospital wards and prior healthcare exposure. Detailed epidemiological information, including patient contact networks, healthcare worker assignments, shared medical equipment, environmental sampling and systematic patient movement data, was not available. Fourth, the pangenome figure was built from unequal numbers of genomes per lineage, the effect of sampling depth on lineage-specific cluster counts was quantified by resampling and is reported in **Supplementary Table 8**. Fifth, the genomes used to place the lineages in a global context were selected for similarity to the study isolates, so the phylogeny resolves the position of ST5994 relative to ST307 but is not a sample of *K. pneumoniae* diversity. Sixth, virulence characterization was based exclusively on genomic prediction, without functional validation, and biofilm production was assessed using a crystal violet assay only. Accordingly, the phenotypic implications of the observed genomic differences require further investigation. Likewise the *fosA* locus was characterized at sequence level only, without expression or enzymatic data, so the mechanistic basis of the fosfomycin difference remains undetermined. Seventh, detailed analyses of clinical outcomes, treatment regimens, infection severity and mortality were beyond the scope of the study. Finally, the FT-IR machine-learning classifier was developed using a relatively small dataset from the same geographical region and is therefore at risk of overfitting, requiring external validation before routine implementation.

## 5 Conclusion

An OXA-48-producing *K. pneumoniae* ST5994 clone was first detected in Cantabria (Spain) in the second quarter of 2023 and, within two years, became one of the two dominant carbapenemase-producing *K. pneumoniae* lineages in the region. To our knowledge this is both the first description of ST5994 in Europe and the first association of this sequence type with OXA-48 anywhere: none of the eight publicly available ST5994 genomes carries a blaOXA-48 gene. Whole-genome phylogeny places ST5994 and ST307 as monophyletic sister clades with maximal support, and the alternative hypothesis that ST5994 arose from within the local ST307 population was rejected: ST5994 is related to ST307 but is not derived from it. Additionally, *K. pneumoniae* ST5994 strains showed comparable multidrug resistance profiles while showing a reduced repertoire of accessory virulence-associated genes, including the absence of ICEKp4 and the *ybt* operon. Surveillance data revealed that *K. pneumoniae* ST5994 rapidly displaced *K. pneumoniae* ST307 and other high-risk clones in our region. The epidemiological and genomic findings were compatible with sustained dissemination within the regional healthcare network rather than a single confined outbreak, although the transmission pathways could not be definitively established.

Pangenome comparisons identified ST-specific adaptations, including T6SS and cell-wall changes (absence of polysaccharide operons in *K. pneumoniae* ST5994), and involving surface structure remodeling, being consistent with bacterial evolution under clinical pressures. Finally, FT-IR spectroscopy coupled with machine learning allowed accurate and rapid discrimination of *K. pneumoniae* ST5994 from other HRCs. This tool results complementary to whole genome sequencing, highlighting its potential as a cost-effective tool for real-time subtyping and infection control.

Overall, our findings highlight the importance of continuous genomic surveillance for the early detection of emerging high-risk *K. pneumoniae* lineages and support the integration of molecular surveillance into infection prevention and control programs. Early recognition of newly emerging clones may also contribute to antimicrobial stewardship by facilitating timely implementation of targeted infection-control measures and improving the epidemiological information available to guide clinical and public health decision-making beyond the study region.

## Data Availability

The 64 whole genome sequences in this study were uploaded to NCBI within BioProject accession PRJNA1378463 and raw sequences are available at NCBI SRA repository with accessions SRR36388020 to SRR36388084 and BioSamples accession numbers ranging from SAMN53838412 to SAMN53838476.

## Supplementary material

Supplementary Figure 1 Minimum spanning tree of *K. pneumoniae* ST307 genome sequences.

Supplementary Figure 2 IncFIB(K) plasmid map comparing both *K. pneumoniae* ST307 and *K. pneumoniae* ST5994 strains.

Supplementary Figure 3 IncL (pOXA-48) plasmid map of both *K. pneumoniae* ST307 and *K. pneumoniae* ST5994 strains.

Supplementary Figure 4 Quantification of biofilm formation by *K. pneumoniae* isolates using the crystal violet (CV) staining method.

Supplementary Figure 5 Fourier-transform Infrared spectra of CP-KP.

Supplementary Figure 6 Maximum-likelihood tree comprising 668 genomes (3 representatives per sequence type).

Supplementary Figure 7 Minimum spanning tree based on cgMLST distances of 1,908 public complete genomes plus 64 from this study.

Supplementary Figure 8 Minimum spanning tree based on cgMLST distances (detail).

Supplementary Table 1 Confusion matrix of the Fourier-transform infrared spectroscopy classifier on the training set.

Supplementary Table 2 List of CP-KP isolates with ST other than ST11, ST23, ST307, ST5000 or ST5994 used in FT-IR analysis.

Supplementary Table 3 K*. pneumoniae* ST5994 isolates used in this study.

Supplementary Table 4 K*. pneumoniae* ST307 isolates used in this study.

Supplementary Table 5 Transmission cluster partition as a function of the single-linkage SNP threshold.

Supplementary Table 6 Edge list of the minimum spanning trees of core-genome SNP distances.

Supplementary Table 7 Publicly available *Klebsiella pneumoniae* ST5994 genomes.

Supplementary Table 8 Dependence of the lineage-specific gene cluster counts on sampling depth.
